# School-age effects of the newborn individualized developmental care and assessment program for preterm infants with intrauterine growth restriction: preliminary findings

**DOI:** 10.1186/1471-2431-13-25

**Published:** 2013-02-19

**Authors:** Gloria McAnulty, Frank H Duffy, Sandra Kosta, Neil I Weisenfeld, Simon K Warfield, Samantha C Butler, Moona Alidoost, Jane Holmes Bernstein, Richard Robertson, David Zurakowski, Heidelise Als

**Affiliations:** 1 Department of Psychiatry, Neurobehavioral Infant and Child Studies, Enders Pediatric Research Laboratories, EN-107, Children’s Hospital Boston, Harvard Medical School, 320 Longwood Avenue, 02115, Boston, MA, USA; 2 Department of Neurology, Developmental Neurophysiology Laboratory, Enders Pediatric Research Laboratories, EN-109-110, Children’s Hospital Boston, Harvard Medical School, 320 Longwood Avenue, 02115, Boston, MA, USA; 3 Department of Radiology, Computational Radiology Laboratory, Main 2, Children's Hospital Boston, Harvard Medical School, 300 Longwood Avenue, 02115, Boston, MA, USA; 4 Department of Radiology, Main South 1, Children’s Hospital Boston, Harvard Medical School, 300 Longwood Avenue, 02115, Boston, MA, USA; 5 Department of Anesthesiology, Perioperative & Pain Medicine, Pavilion 121, Children’s Hospital Boston, Harvard Medical School, 300 Longwood Avenue, 02115, Boston, MA, USA

**Keywords:** Newborn individualized developmental care and assessment program (NIDCAP), Intrauterine growth restriction (IUGR), Preterm, School-age, Executive function, Memory, Spectral coherence, MRI tissue segmentation, Cerebellum, Rey-Osterrieth Complex Figure Test

## Abstract

**Background:**

The experience in the newborn intensive care nursery results in premature infants’ neurobehavioral and neurophysiological dysfunction and poorer brain structure. Preterms with severe intrauterine growth restriction are doubly jeopardized given their compromised brains. The Newborn Individualized Developmental Care and Assessment Program improved outcome at early school-age for preterms with appropriate intrauterine growth. It also showed effectiveness to nine months for preterms with intrauterine growth restriction. The current study tested effectiveness into school-age for preterms with intrauterine growth restriction regarding executive function (EF), electrophysiology (EEG) and neurostructure (MRI).

**Methods:**

Twenty-three 9-year-old former growth-restricted preterms, randomized at birth to standard care (14 controls) or to the Newborn Individualized Developmental Care and Assessment Program (9 experimentals) were assessed with standardized measures of cognition, achievement, executive function, electroencephalography, and magnetic resonance imaging. The participating children were comparable to those lost to follow-up, and the controls to the experimentals, in terms of newborn background health and demographics. All outcome measures were corrected for mother’s intelligence. Analysis techniques included two-group analysis of variance and stepwise discriminate analysis for the outcome measures, Wilks’ lambda and jackknifed classification to ascertain two-group classification success per and across domains; canonical correlation analysis to explore relationships among neuropsychological, electrophysiological and neurostructural domains at school-age, and from the newborn period to school-age.

**Results:**

Controls and experimentals were comparable in age at testing, anthropometric and health parameters, and in cognitive and achievement scores. Experimentals scored better in executive function, spectral coherence, and cerebellar volumes. Furthermore, executive function, spectral coherence and brain structural measures discriminated controls from experimentals. Executive function correlated with coherence and brain structure measures, and with newborn-period neurobehavioral assessment.

**Conclusion:**

The intervention in the intensive care nursery improved executive function as well as spectral coherence between occipital and frontal as well as parietal regions. The experimentals’ cerebella were significantly larger than the controls’. These results, while preliminary, point to the possibility of long-term brain improvement even of intrauterine growth compromised preterms if individualized intervention begins with admission to the NICU and extends throughout transition home. Larger sample replications are required in order to confirm these results.

**Clinical trial registration:**

The study is registered as a clinical trial. The trial registration number is NCT00914108.

## Background

Experience in the newborn intensive care unit (NICU) alters development [1,2]. Of the 4.3 million annual US births, 12.7% are premature [3], of these 30% are intrauterine growth restricted (IUGR) [4]. IUGR preterms show increased mortality and morbidity, brain changes and developmental disabilities (>50%) [4,5]. The Newborn Individualized Developmental Care and Assessment Program (NIDCAP) [6] reduces neurodevelopmental NICU-sequelae for appropriately grown for gestational age (AGA) preterm infants [1,2,7-13]. Beneficial effects were also recently demonstrated for IUGR preterm infants [14]. Long-term effectiveness of NIDCAP into school-age so far has been demonstrated only for AGA preterm children [15,16]. The current study examined the hypothesis that NIDCAP is also effective in improving school-age outcome for IUGR preterm-born children in terms of executive function (EF), as well as of occipital to frontal coherence (EEG), and frontal and cerebellar brain tissue volumes when compared to IUGR preterm-born children who received standard NICU care.

With advances in perinatology and neonatology survival rates not only of preterm infants appropriately grown for gestational age (AGA) [17], but also of severely IUGR preterm infants [18] have significantly increased. The added burden of intrauterine growth restriction in the prematurely born infant is associated with considerably higher cost not only in terms of NICU care but also in terms of long-term educational and social resource requirements when compared to those required by the AGA preterm [4]. Research suggests that as IUGR preterms mature and both academic and life challenges increase in complexity, the gap between them and their full term and AGA preterm peers widens on many measures of cognition, educational achievement, and especially executive function [19-22]. Preterm-born children in general and especially IUGR preterms are over-represented among those requiring early intervention and special education services. As the demands of the learning environment become steadily more complex, such children typically require increased educational services [23]. The impact of such children’s alternative neuropsychological development on IQ and learning capacities intensifies in the face of increased demands on abstract integrative abilities. School-age IUGR preterm-born children are characterized by a range of mental control and executive function difficulties that include poor planning, problem solving, and impaired inhibitory controls, all of which are core abilities associated with poor educational performance [24,25]. The lifetime costs associated with IUGR prematurity are not only those of the newborn intensive care they must receive but include also increased costs for educational and social resources [4,26].

While some of the integrative developmental difficulties might be explained by the cumulative effect of medical complications such as temperature control and glucose metabolism instabilities inevitably associated with preterm birth [27], the infant’s sensory experience in the Newborn Intensive Care Unit (NICU) environment with exposure to bright lights, high sound levels, and frequent stressful interventions exerts additional harmful, if not damaging effects on the dysmature brain, and thus further alters its subsequent development [5,14,28,29]. The extrauterine environment appears to present an additional challenge for the already compromised brain of the IUGR preterm infant during a very sensitive period of rapid growth and differentiation. The goal of the NIDCAP intervention is to mitigate the sensory impact of the NICU environment. It is based on the assumption that all infants, no matter how small, display reliably observable behaviors in the form of autonomic and visceral responses, motor system patterns, and state behaviors [30-32], which express the current appropriateness for the individual infant of the environment (sound, light, activity, affective climate), as well as of the timing and quality of all caregiving and all social interactions [6,32,33]. Each infant’s behavior in turn may aid the caregiving professionals in the reliable identification of the infant’s restfulness, comfort and well-being, as well as the infant’s impending stress responses. Monitoring of the infant’s behavior allows for ongoing provision of care in a manner that is matched to the infant’s tolerance level, thus minimizing additional stressors on an already burdened system.

The NIDCAP approach has been demonstrated to be associated with improved longer-term outcomes in AGA preterm-born children in one study at age 5 years, with significantly fewer severe disabilities [15] and another study at age 8 years with significantly better visual-motor function and improved EEG spectral coherence, in the form of more effective connections between occipital and frontal lobes [16] in the NIDCAP compared to the control group. The current study extends this work by examining for the first time the effectiveness of NIDCAP interventions on neuropsychological function, cortical coherence and brain structure at school-age in the IUGR preterm-born population.

## Methods

### Design

Follow-up evaluations at nine years (y) of age corrected for prematurity (CA) were conducted for the IUGR preterm-born children of a two-group (control - C and experimental - E) randomized controlled trial (RCT). The original study, reported elsewhere, [14] and the study protocol for the school-age follow-up were approved in full compliance with the Helsinki Declaration by the institutional review board for research with human subjects at the study hospital. All outcome assessment personnel were purposefully kept blinded to the children’s group assignments throughout the study.

### Subjects

The original RCT sample [14] consisted of 30 preterm infants with severe IUGR and their parent(s). Recruitment extended from January 1996 – May 2000; seventy-five infants and their parents met study criteria. Of these, 45 were approached and 30 families signed consent. They and their infants were randomized to control (C) (standard care in the study hospital at the time of study) and experimental (E) care (NIDCAP), yielding 18 C- and 12 E-group subjects. Non-participating eligible infants and their families were comparable to those participating in all background criteria. All infants were recruited from the NICU of a large tertiary care academic center in an urban US setting (>7000 births/year). The NICU was a 48-bed level-III unit that served almost exclusively newborns delivered at the same hospital. IUGR was defined as birth weight and head circumference < 5^th^ percentile (%ile) for GA and due to placental insufficiency as documented by two or more of the following: Maternal high blood pressure, oligohydramnios, and/or absent or compromised Doppler-diagnosed end diastolic flow velocity. All deliveries were initiated due to the fetus’ severe growth restriction.

Selection criteria included: For the mother - residence in the greater urban area surrounding the study hospital; age ≥14 years; absence of major maternal medical/psychiatric illness, chronic medication treatment, and history of substance abuse; telephone accessibility; and some English language facility. For the infant these included - inborn at study hospital; GA 29 - 33w by estimated date of confinement (EDC) based on 2^nd^ or 1^st^ trimester fetal ultrasound, mother’s dates and/or Ballard GA assessment [34]; < 5^th^ percentile (%) in birthweight and head circumference for GA (Gairdner and Pearson growth charts) [35]; singleton or twin with AGA sibling; 5-minute Apgar ≥ 5; absence of major chromosomal or congenital anomalies, congenital infections, or diagnosed prenatal brain lesions; and deemed viable by the attending neonatologist [14].

**Figure 1:**
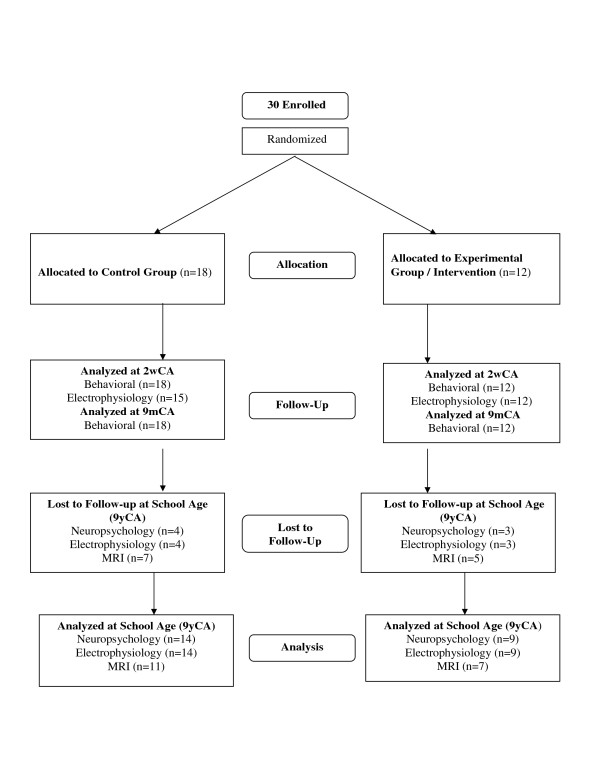
Consort flow chart.

The 30 (18C, 12E) original study families were re-contacted once the children reached nine years (y) corrected age for prematurity (CA). Three of the families were not located; four declined participation. Of the original 30 study infants’ families, 23 (14C; 9E) agreed to participate in the follow-up study and signed a new institution-approved consent form prior to data acquisition. Background comparability of the participating school-age children and those not participating was examined in terms of newborn medical, anthropometric and demographic background variables. Comparability of the participating school-age C- and E-group children was assessed in terms of their newborn anthropometric, medical and demographic background variables as well as in terms of school-age anthropometric, medical and schooling background and their mothers’ IQ. Figure 1 shows the Consort Flow Chart of the school-age follow-up study (Figure 1).

### Control and experimental group experience

C-group infants received the standard care practiced at the time of study [14]. This included effort at primary care nursing, variable staff-dependent parent inclusion, shielding of incubators, use of infant clothing, side and foot rolls and pacifiers, and variable staff-dependent encouragement of skin-to-skin holding and breast-feeding. Contamination effects from E- to C-group infants were not prevented systematically nor measured, e.g. a nurse involved in the care of an E-subject might also provide care to a C-subject on an occasional basis. Any E-effects found, therefore, should be considered conservative since by definition they were in excess of possible C-group contamination.

The E-group received the NIDCAP intervention [6], which emphasized each infant’s behavioral individuality. The E-group’s NIDCAP team consisted of a developmental psychologist and a pediatrician, both certified NIDCAP Professionals in keeping with the NIDCAP Federation International’s (NFI) certification standards [6] and experienced in the implementation of the NIDCAP intervention. They provided weekly detailed infant observations and daily contact with the E-group’s professional care providers (nurses, neonatologists, physio- and respiratory therapists, and social workers) in the NICU and with the parents. Their role was to support caregivers and parents in their understanding of the infants’ stress and comfort signals, and to adjust their care accordingly in order to ensure continuity and consistency of individualized care for each E-group infant. Weekly formal behavioral observations translated into neurobehavioral reports and daily (seven days/week) support to individualized caregiving began with initial stabilization and ended at 2 weeks corrected age (2wCA) [14].

### Newborn medical, anthropometric and demographic background measures; school-age anthropometric and health measures, and mothers’ IQ

Newborn medical, anthropometric and demographic information available in a database from the infants’ previous evaluations was accessed for the school-age study sample. These data were used to compare the participating to non-participating children, as well as to assess comparability between C- and E-group children at school-age. Anthropometric, medical and academic history measures at school-age were obtained by direct measurement in terms of weight, height and head circumference as well as in terms of growth percentiles for corrected age, and by parent interview [36]. It was hypothesized that the school-age participants and the non-participants would be comparable in medical and demographic background measures. It was furthermore hypothesized that the C- and E-group children at school-age would be comparable in newborn medical and demographic, and in school-age anthropometric, medical and academic history measures.

Since the literature reports the importance of parent IQ for child school-age functioning [37,38], mothers’ IQ was assessed at the school-age study point with the Kaufman Brief Intelligence Test, Second Edition (KBIT-2) [39]. This test yields three standardized scores, each with a mean (X) of 100 and a standard deviation (SD) of 15, namely a Verbal IQ, a Non-Verbal IQ, and an IQ Composite. Confidentiality was assured prior to administration of the IQ measure, which was performed by a skilled examiner in a private comfortable testing room.

It was hypothesized that C-and E-groups at school-age would be comparable in parent IQ. It was also hypothesized that the children’s school-age cognitive function might be correlated with parent IQ, in which case all outcome measures would have to be corrected for parent IQ. On the basis of the earlier AGA school-age NIDCAP outcome study [16] significant effects favoring the IUGR-preterm E-group school-age children were hypothesized specifically for neuropsychological functioning in terms of executive function (EF) including visual-motor and memory aspects. On the basis of the earlier AGA spectral coherence findings at school-age [16] as well as IUGR spectral coherence findings in the newborn period [14,40] it was hypothesized that the electrophysiological functioning effects would favor the E-group in terms of increased spectral coherence between long-distance frontal and occipital brain regions as well as decreased widely distributed coherences of short and medium distances. Neurostructural effects reflected in improved frontal white matter and cerebellar volumes for the E-group were hypothesized on the basis of the current literature [41-47].

### Anthropometric, medical and schooling history measures at school-age

All children were measured at 9 years corrected age (9yCA) in terms of weight, height and head circumference as well as in terms of growth percentiles for corrected age. A brief medical and academic history was obtained by parent interview.

### Neuropsychological outcome measures at 9 years corrected age

Due to the study’s small sample size an abbreviated set of neuropsychological measures was designed and administered. The testing set included an IQ measure, namely the Kaufman Assessment Battery for Children, Second Edition (KABC-II) [48] which yields three main Index scores (

$$\bar{X}$$

= 100; SD = 15), namely a Mental Processing Index (MPI), a Sequential Scale Index, and a Simultaneous Scale Index; a school achievement measure, namely the Woodcock-Johnson III Tests of Achievement (WJ-III) [49] with the Standard Scores (

$$\bar{X}$$

= 100; SD = 15) of the Broad Reading Cluster (Letter/Word Identification, Reading Fluency, Passage Comprehension), and the Academic Skills Cluster (Letter/Word Identification, Calculation, Spelling); and an untimed measure of executive function, the Rey-Osterrieth Complex Figure Test (ROCFT) [50-52], which assesses integrative gestalt planning abilities, perceptual organization, visual motor planning and visual memory in three conditions, the Copy condition, the Immediate Recall and the Delayed Recall conditions (20 minutes after the original copy condition). The Developmental Scoring System (DSS-ROCF) [53] yields for each of the three conditions three mutually exclusive scores, an Organization score, a Structural Elements Accuracy score, and an Incidental Elements Accuracy score. These nine scores were utilized. All have been demonstrated to be sensitive to prematurity [53-57]. The *Organization* score provides a measure of the child’s appreciation of the basic structure of the design. It ranges from 1 (poorly organized) to 13 (well organized). The *Structural Elements Accuracy* score quantifies the number of line segments (0 to 25) reproduced from the ROCF Base Rectangle (BR) and Main Structures (MS). The *Incidental Elements Accuracy* score quantifies the number (0 to 39) of Outer Configuration and Internal Detail segments.

All neuropsychological assessments were performed by an experienced neuropsychologist, purposefully blinded as to the subjects’ group identities. All studies were performed at a specially designed neurobehavioral laboratory outfitted with a one-way mirror window and two hidden cameras; the scoring of all assessments was double-checked for accuracy. Parent(s), if they so chose, and their children agreed, watched the assessments through a one-way-mirror window or were in the room with their child. Rest breaks were interspersed as indicated. All neuropsychological variables derived from the assessments were coded and double-checked by one of two experienced blinded coders.

### Neurophysiological outcome measures at school-age

Following the neuropsychological assessment, the children were assessed neurophysiologically (EEG) in the Eyes Closed (ECL) alert state. ECL was chosen for analysis since eye movement contamination tends to be minimal in this condition. Paroxysmal epochs of eye movement, muscle activity, and whole body movement were visually identified by the senior neurophysiologist and excluded from subsequent analysis. ECL EEG was collected in 2-minute-segments for a total of 12 minutes by a registered EEG technologist with expertise in pediatric EEG. Both the neurophysiologist and technologist were blinded as to the subjects’ study and group identities. All neurophysiologic assessments were performed in a specially designed research EEG suite immediately adjacent to the neurobehavioral testing laboratory. The parent(s), if they chose and their children agreed, watched the EEG assessments through a one way-mirror window within the EEG suite and/or sat with their child in the EEG booth within the suite.

**Figure 2:**
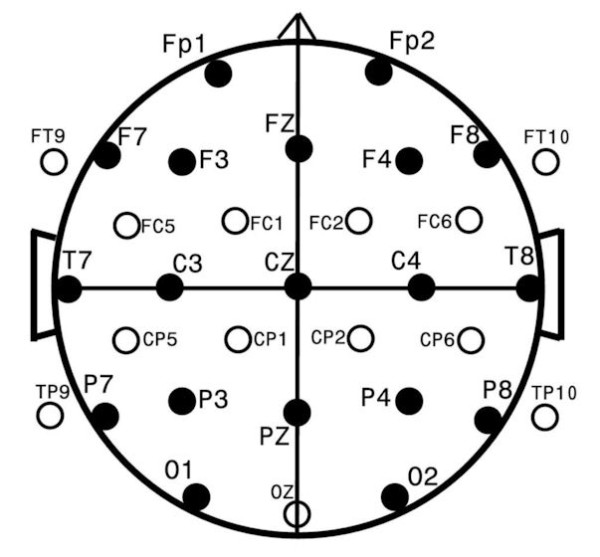
**Standard EEG electrode names and positions.** Head in vertex view, nose above, left ear to left. EEG electrodes: Z: Midline: FZ: Midline Frontal; CZ: Midline Central; PZ: Midline Parietal; OZ: Midline Occipital. Even numbers, right hemisphere locations; odd numbers, left hemisphere locations: Fp: Frontopolar; F: Frontal; C: Central; T: Temporal; P: Parietal; O: Occipital. The standard 19, 10–20 electrodes are shown as black circles. An additional subset of 17, 10–10 electrodes are shown as open circles.

EEG data were collected from 32 EEG channels at a 256 Hz sampling rate with subsequent bandpass filtering from 1–50 Hz and a 60 Hz notch filter to minimize environmentally induced artifacts such as data contamination from nearness to electrical power mains. Figure 2 shows the standard EEG electrode names and positions. (Figure 2) Analyses were based upon data placed in the Laplacian reference-electrode-free format which is primarily sensitive to underlying cortex, relatively insensitive to deep/remote EEG sources, and unrelated to the particular reference used during data collection [58]. Remaining eye blink and eye movement artifacts, which may be surprisingly prominent even during the eyes closed state, were removed from EEG by utilizing the source component technique [59,60] as implemented in the BESA™ software package. Spectral analysis, including spectral coherence calculation as outlined by van Drongelen [61], was performed using a Nicolet™ software package. Using a spectral resolution of 2 Hz per data point (16 points over 32 Hz), among all 32 channels, 4416 individual coherence variables were created. A multivariate regression analysis, originally described by Semlitsch [62], was used which, by first identifying a signal proportional to a known source of artifact (e.g., prefrontal slow delta for eye blink artifact and fast beta for temporal muscle artifact) effectively removes remaining, low amplitude, artifactual contributions. As previously described, these coherence variables – now largely free from artifact - were reduced in number by using in-house-developed [63] principal components analysis (PCA) software that includes Varimax rotation and is suited to factoring of large asymmetrical matrices. Forty coherence factors, previously created on an independent age-comparable normal control group (n = 219), and reflecting 48% of total coherence variance [64,65] were formed on the current school-age subjects utilizing the previous principal components analysis-generated rule. Given the sample size the first twenty factors were utilized in the subsequent analyses.

### Neurostructural outcome measures at school-age

**Figure 3:**
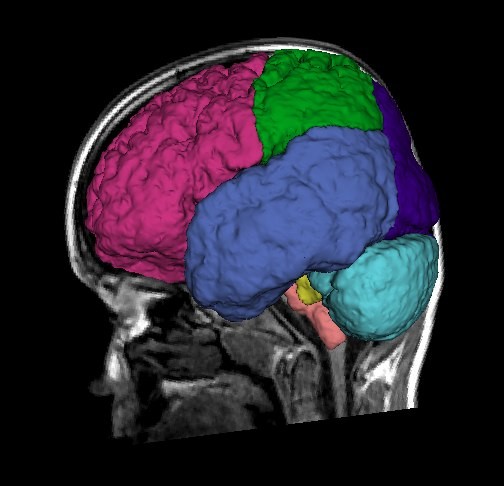
**3D rendering of parcellation of a 9-year-old child’s brain from MRI.** The surface model is depicted on top of a mid-sagittal slice from the T1-weighted MRI. Comparison of regional tissue volumes across subjects can be employed to indicate localized structural differences.

**Figure 4:**
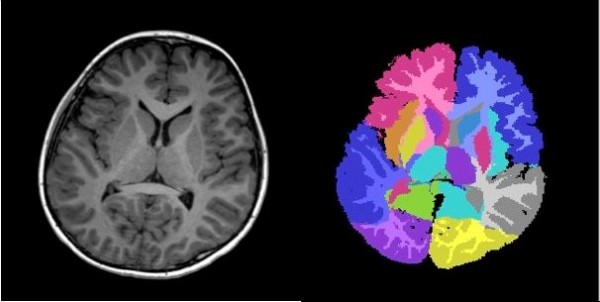
**Demonstration of T1-weighted MRI tissue segmentation combined with parcellation of a 9-year-old child’s brain.** Axial slice on the left; corresponding combined tissue segmentation/parcellation on the right.

While EEG is sensitive to temporal aspects of neural function, measures the electrical consequences of neural activity, and provides surface summaries, MRI specifies location and type of underlying brain structural differences. Non-invasive 3D-MRI of brain structure was used in order to test for NIDCAP-supported brain structural improvement at school-age and to evaluate the assumption of neurodevelopmental continuity to school-age. Data were acquired at 3Tesla (Siemens Tim Trio, Siemens, Erlangen, Germany) with an MR imager using a 32-channel head coil. High spatial resolution echo-planar diffusion weighted images were acquired (24 cm FOV, matrix 128x128, 2 mm thick contiguous slices). Geometric distortion from magnetic susceptibility differences was minimized with a short echo time (TE = 78 ms) and parallel imaging (iPAT 2). Thirty b = 1000 s/mm2 images were acquired at directions evenly spaced on the sphere along with five baseline (b = 0) images. Volumetric tissue segmentation was utilized. MRI tissue volumes were measured in cubic millimeters, per structure. To control for ventricular size, all tissue measures were expressed as a percentage of total parenchyma. White matter (WM) was identified for the right and left frontal lobes, and percent of total tissue volumes were measured for the right and left cerebellum for the test of the primary hypothesis, which stated that frontal lobe (prefrontal inclusive of pre-motor and sensorimotor) white matter and cerebellum volumes would differentially favor the E-group and support hypothesized improved EF. Frontal lobe volumes are widely reported of key importance for executive and associated integrative functions. The sensitivity of the cerebellum has been highlighted more recently: Cerebellar structures develop rapidly between 29 and 33 weeks gestation - the corresponding high metabolic rate makes them particularly vulnerable to trophic disturbances, and they have wide-ranging interconnectivity with many higher cortical areas well beyond the traditionally assumed motor function area, implicating them in various meta-cognitive function abnormalities in the preterm born child [41-46] and more recently implicated in the cerebellar cognitive affective syndrome (CCAS) [66] which includes impairments in executive and visual spatial functions. The primary MRI outcome measures of bilateral frontal (F) WM and bilateral cerebellar inclusive of vermis tissue volumes were supplemented on a purely exploratory basis by additional descriptive brain structure tissue volume measures, given the relative dearth of information regarding specificity of involvement of brain regions in the IUGR preterm condition at school-age. Given the early prenatal brain insult involved in IUGR, the exploration attempted to cast a wide net. Additional exploratory measurements included the tissue volumes of bilateral frontal grey matter (GM), as well as bilateral WM and GM of occipital, temporal and parietal regions, total tissue volumes of brainstem inclusive of the ventral diencephalon, right and left thalamus, nucleus accumbens, caudate, putamen, hippocampus, and amygdala, as well as the insula. Post-acquisition MRI processing techniques were used for tissue segmentation and 3D renderings with advanced volume visualization and quantification as described and utilized elsewhere [67,68]. Regional tissue volumes were defined by warping an anatomical template onto each subject’s data using non-rigid registration algorithms [68]. Figure 3 shows the regional parcellation schema utilized and Figure 4 the tissue segmentation strategy employed.

### Data analysis

All statistical analyses were performed using BMDP 2007™ software [69]. Continuous variables were submitted to univariate analysis of variance (ANOVA) (BMDP7D) [70]. In cases of unequal variance, the Browne-Forsythe test of variance (F*) was used. Categorical variables were submitted either to Fisher’s exact probability test (FET) for 2 x 2 tables; or to the Pearson chi-square (χ^2^) test for all other multiple row by column variable arrays [69,71]. For all analyses, two-tailed values of p < 0.05 were considered statistically significant. The sample sizes provided 80% power to detect large effects in the outcome measures between the two groups, generally effect sizes >1.0 [72].

Stepwise discriminate analysis (DSC) (BMDP7M) was employed for the school-age EF, the electrophysiological and the neurostructural domains. Wilks’ lambda [73] and jackknifed [74,75] classification were performed in order to ascertain two-group classification success per domain and across domains at school-age outcome. Jackknifed classification efficiency is calculated by sequentially eliminating one case at a time, computing the discriminant function based on remaining subjects, and using the resultant discriminant to classify the eliminated case. Percent correct classification is based on the overall success of the total sample’s cumulative classification in this manner. Canonical correlation analysis (BMDP6M) was used in order to explore the relationships among the EF, electrophysiological and neurostructural domains at school-age, as well as the neurobehavioral data domain relationship at 2wCA (Assessment of Preterm Infants’ Behavior (APIB) [76] and school-age (Rey-Osterrieth Complex Figure Test [50,53]).

## Results

### Newborn background comparability of participating and non-participating school-age subjects, and of control and experimental group subjects at school-age

The children participating in the school-age study (n = 23) were comparable in anthropometric, medical and demographic background variables at birth to the children who did not participate (n = 7). In addition, the participating school-age control (C = 14) and experimental (E = 9) groups were comparable on all newborn medical, anthropometric and demographic background variables.

### School-age growth and medical measures

The school-age control and experimental groups were comparable in age at neuropsychological assessment {

$$\bar{X}$$

years (SD)} {C: 9.63 (0.92); E: 9.76 (1.10); p = 0.77}. All EEG and MRI studies were performed within a week of the neuropsychological assessment. Weight/weight percentiles, height/height percentiles and head circumference/head circumference percentiles were comparable between the two groups, as were health outcomes measured at school-age such as hearing loss, diagnosed disabilities, and utilization of special services (Table 1).

**Table 1:** **Anthropometric, medical and demographic variables at time of evaluation**^**a**^

**Variable**	**Control (C)** **(n = 14)**	**Experimental (E)** **(n = 9)**	***p***
Metric			
Weight, kg	36.16 (12.03)	32.69 (7.39)	0.41
Height, cm	138.84 (9.75)	136.58 (10.90)	0.63
Head Circumference, cm	53.21 (1.83)	52.11 (2.03)	0.21
Percentiles			
Weight percentile	60.00 (35.30)	55.78 (31.16)	0.77
Height percentile	57.69 (29.25)	48.22 (38.00)	0.54
Head Circumference percentile	61.46 (29.96)	45.11 (32.26)	0.25
Age at testing, years	9.63 (0.92)	9.76 (1.10)	0.77
Mother’s IQ			
Verbal	109.84 (20.49)	102.33 (11.11)	0.27
Non-Verbal	111.24 (14.88)	104.67 (12.06)	0.26
Composite	111.16 (16.75)	103.44 (11.45)	0.20
Male/female	9/5	4/5	0.42^b^
Special school services, yes/no	10/4	8/1	0.61^b^
Disability Diagnoses, yes/no	6/8	5/4	0.68^b^
Hearing loss, yes/no	1/13	0/9	1.0^b^
Mother’s Education Level (HS/College/Grad)	4/7/3	4/3/2	0.69^c^
Income (<50 K/50-75 K/>75 K)	1/2/11	2/1/6	0.58^c^
Ethnicity (Caucasian/Black/Hispan/Other)	10/1/1/2	7/1/0/1	0.85^c^

^a^Results are means and (standard deviations) unless otherwise noted. Statistical analyses used are Brown-Forsythe Univariate Analysis of Variance: F*, ^b^Fisher’s exact Test, and ^c^Pearson’s chi square: χ^2^. *p* = probability.

### Parent IQ and its relationship to child function at school-age

As expected, the mothers’ IQ (Mother IQ) was significantly correlated with their children’s mental functioning. (Mother Verbal IQ × Child Mental Processing Index: r = 0.527; p < 0.02; Mother Verbal IQ × Child Simultaneous Scale Index: r = 0.515; p < 0.02; Mother Non-Verbal IQ × Child Mental Processing Index: r = 0.653; p < 0.001; Mother Non-Verbal Index × Child Simultaneous Scale Index: r = 0.742; p < 0.001; Mother IQ Composite x Child Mental Processing Index: r = 0.644; p < 0.01; Mother IQ Composite × Child Simultaneous Scale Index: r = 0.657; p < 0.001.) (Of interest was that Child Sequential Scale Index correlated neither with Mother Verbal IQ, Mother Non-Verbal IQ nor Mother IQ Composite in this sample.) Given the high correlations all neurodevelopmental outcome measures were statistically corrected for the mothers’ Verbal IQ and Non-Verbal IQ by use of Partial Correlation and Multivariate Regression Analysis (BMDP6M). The mothers’ Verbal IQ, Non-Verbal IQ and IQ Composite, while not significantly different between the C- and E-groups, showed a trend in the direction of higher IQ scores in the C-group as compared to the E-group {Verbal IQ: C:109.84 (20.49); E:102.33 (11.11); p = 0.27, Non-Verbal IQ: C: 111.24 (14.88); E: 104.67 (12.06); p = 0.26; IQ Composite: C: 111.16 (16.75); E: (103.44 (11.45); p = 0.20}.

### Neuropsychological outcome at school-age

All twenty-three (14C; 9E) returning school-age subjects completed their neuropsychological test protocols. C- and E-groups were comparable in overall cognitive function as measured with the K-ABC-II [48] Indices: Mental Processing Index: C: 104.23 (20.44); E: 99.76 (12.08); p = 0.52; Sequential Scale Index: C: 98.80 (17.67); E: 98.87 (13.32); p = 0.99; and Simultaneous Scale Index: C: 99.84 (17.62); E: 98.69 (14.04); p = 0.86. Their performance was comparable on academic achievement measures: Woodcock-Johnson III Tests of Achievement (WJ-III) [49] Broad Reading Cluster (Letter/Word Identification, Reading Fluency, and Passage Comprehension): C: 102.92 (26.79); E: 103.12 (11.13); p = 0.98; Academic Skill Cluster (Letter/Word Identification, Calculation, and Spelling): C: 105.13 (31.47); E: 105.81 (11.60); p = 0.94. (Table 2). All K-ABC-II and WJ-III group mean scores were within normal limits. Standard deviations of cluster and subtest scores consistently were larger for the C (minimum WJ-III, 17.60 - maximum WJ-III, 31.47; minimum KABC, 17.60 - maximum KABC, 21.11) than E-group (minimum WJ-III, 7.20 - maximum WJ-III, 13.50; minimum KABC, 6.57 - maximum KABC, 14.0) perhaps indicating the intervention’s effect of reducing extremely poor scores.

**Table 2:** Woodcock-Johnson III Tests of Achievement

**Variable**	**Control** **(n = 14)**	**Experimental** **(n = 9)**	***p***
Word Identification	105.59 (27.84)	106.41 (13.50)	0.93
Reading Fluency	105.20 (17.60)	100.13 (11.53)	0.41
Math Calculation	101.01 (19.76)	97.21 (8.76)	0.54
Spelling	103.25 (33.71)	107.95 (12.72)	0.64
Passage Comprehension	97.73 (23.22)	102.43 (7.20)	0.49
Broad Reading	102.92 (26.79)	103.12 (11.13)	0.98
Academic Skills	105.13 (31.47)	105.81 (11.60)	0.94

Results are means (SD). Mean = 100; standard deviation 15. Statistical analysis used is Brown Forsythe Univariate Analysis of Variance F*, two-tailed.

**Figure 5:**
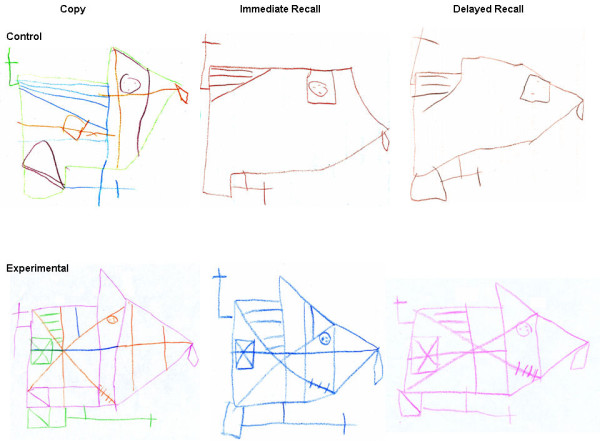
**Rey-Osterrieth complex figure.** The figure represents sample drawings from two study children, one from the Control group, a 9 year 11 month old born at 29w GA; and one from the Experimental group, a 9 year 6 month old born at 32w GA. The conditions displayed are from left to right: Copy, Immediate Recall and Delayed Recall.

When examining the nine mutually exclusive EF scores derived from the DSS-ROCF [53] Copy condition Incidental Accuracy score, significantly favored the E-group over the C-group (p < 0.05), indicating that the E-group children represented the internal features of the figure with significantly greater accuracy than did the C-group children. Two other ROCFT scores showed a trend in favor of the E-group children. These included the Copy condition Organization score (p < 0.15), and the Delayed Recall Structural Accuracy score (p < 0.16). Eleven of the 15 ROCFT scores showed better mean scores for the E- than the C-group, two were near identical in the two groups (Table 3). Overall, the results supported the study’s primary hypothesis that the E-group children would show better EF than the C-group children at school-age. Figure 5 shows sample drawings from two study children, one from the C- and one from the E- group. The lack of significant gain in overall cognitive function and academic achievement by the E-group children may reflect the small sample size. However, differences in sensitivity of population-standardized measures (as exemplified in the K-ABC and the WJ-III) to biological variables, as compared with developmentally-referenced measures (as exemplified by the ROCF), has been noted previously [55,77].

**Table 3:** Rey-Osterrieth Complex Figure Test

**Variable**	**Control** **(n = 14)**	**Experimental** **(n = 9)**	***p***
Copy Basal Level	1.87 (0.78)	2.42 (0.89)	0.15
Copy Organization Score	4.88 (2.79)	6.41 (2.64)	0.20
Copy Style Score	2.19 (1.05)	2.26 (1.22)	0.90
Copy Structural Accuracy Score	21.66 (6.66)	24.08 (1.75)	0.21
Copy Incidental Accuracy Score	32.07 (10.72)	38.22 (1.66)	0.05
Immediate Recall Basal Level	1.96 (0.98)	1.95 (0.52)	0.95
Immediate Recall Organization Score	4.48 (3.02)	4.69 (1.54)	0.83
Immediate Recall Style Score	2.59 (1.42)	2.53 (1.21)	0.92
Immediate Recall Structural Accuracy Score	15.27 (7.65)	17.03 (4.13)	0.48
Immediate Recall Incidental Accuracy Score	23.23 (8.34)	23.20 (7.84)	0.99
Delayed Recall Basal Level	2.30 (1.03)	2.09 (0.86)	0.61
Delayed Recall Organization Score	5.69 (3.39)	5.15 (2.43)	0.66
Delayed Recall Style Score	2.26 (1.38)	2.93 (1.09)	0.21
Delayed Recall Structural Accuracy Score	15.38 (9.19)	19.19 (2.53)	0.16
Delayed Recall Incidental Accuracy Score	23.00 (8.60)	24.77 (7.39)	0.60

Results are means (SD). Statistical analysis used is Brown-Forsythe Univariate Analysis of Variance F*, two-tailed.

In order to ascertain two-group classification success for the EF measures discriminant function analysis utilizing the 9 ROCFT-measures identified 3 measures (Incidental Accuracy in the Copy condition, Incidental Accuracy in the Immediate Recall condition, and Structural Accuracy in the Copy condition). These showed a trend toward differentiating the C- from the E-group. (Table 3) Jackknifed classification success utilizing these 3 measures, showed 73.9% correct subject classification [74,75] (Wilks’ Lambda = 0.7234; df = 3,19; p = 0.10) (Table 4). Misclassified were 2 C- and 4 E-subjects.

**Table 4:** Discriminant function analysis of Rey-Osterrieth measures

Jackknifed Classification* Matrix ***Copy Incidental Accuracy, Immediate Recall Incidental Accuracy, Copy Structural Accuracy*)**	**Correct Classification**	***Control*** **(n = 12)**	**Experimental** **(n = 11)**
Control (C) Group	71.4%	10	4
Experimental (E) Group	77.8%	2	7
Total	73.9%	12	11

Wilks’ Lambda = 0.7234; *df* = 3,19; F = 2.42; *p =* 0.10. Jackknifed classification* efficiency is calculated by sequentially eliminating one case at a time, computing the discriminant function based on remaining subjects, and using the resultant discriminant to classify the eliminated case. Percent correct classification is based on the overall success of the total sample’s cumulative classification in this manner.

### Neurophysiological outcome at school-age

**Figure 6:**
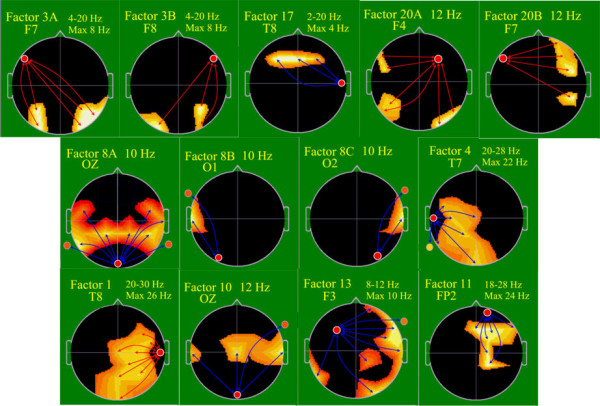
**EEG spectral coherence factors at school-age, control (C) (n =12), experimental (E) (n = 9).** Head shown in vertex view, nose above, left ear to left. EEG frequency and coherence electrodes shown above head. Arrow color illustrates experimental group coherence; green = decreased, red = increased.

Of the 23 subjects 21 (12 C; 9 E) children had complete neurophysiological assessment. Two control group children were too hesitant to participate. The group difference analysis for the first 20 EEG spectral coherence factors showed that Coherence Factor 3 significantly favored the E- over the C-group (p < 0.03) (Figure 6). Factor 3 indicated significantly increased and surprisingly symmetrical, bi-hemispheric, across midline, long-distance coherence for the E-group connecting the frontal left and bilateral occipital-parietal brain regions as well as the frontal left and bilateral occipital-parietal regions in a very broad frequency band (4 – 20 Hz; theta, alpha and slow beta) with maximum frequency at 8 Hz (alpha). This indicates significantly more mature and effective cortical coupling between these distant brain regions [16,78], whose connectivity suggest implications in EF, visual-spatial, visual planning and memory capacities, necessary for effective ROCF production. One other factor, Coherence Factor 17, showed a trend in the direction of lower coherence for the E- than C-group children (p < 0.11). Factor 17 indicated E-group decreased medium-to-short distance coherence between temporal right and midline posterior frontal regions, suggesting decreased cortical coupling between these regions for the E-group. This would be consistent with reduction of short distance over-coupling between regions, which by school-age are expected to function differentially. The persistence of over-coupling in the C-group may well reflect the effect of IUGR-related pathology [14,40].

Discriminant function analysis utilizing the first 20 of the 40 EEG spectral coherence factors identified 8 coherence factors, which significantly differentiated the C- from the E-group children (Table 5). Jackknifed classification success utilizing the 8 factors showed 90.5% correct subject classification [74,75] (Wilks’ Lambda = 0.1561; df = 8,12; p = 0.001). The factors involved included again Coherence Factor 3 (see above for interpretation), and in addition Coherence Factor 20, a bilateral long distance factor with increased connectivity for the E-group between the right and the left frontal areas and the right and left occipital and posterior parietal areas, maximally at 12 Hz (alpha), again implicated in visual-spatial and EF functions [16,78]; Coherence Factor 8, with decreased short distance coupling in the E-group between the occipital and broad central and bilateral parietal/temporal areas maximally at 8 Hz (slow alpha), indicating reduction in likely pathological over coupling [14,40]; Coherence Factor 4, with decreased, relatively short distance, left-hemispheric coherence between the left temporal and left occipital regions at 20 – 28 Hz (slow beta), maximally at 22 Hz, likely indicating reduction in pathological over coupling between the left mid temporal regions and broadly other left hemisphere regions [14,40]; Coherence Factor 1, - almost in mirror image of Factor 4, but now with increased coherence in the E- compared to the C-group, between right mid temporal and broad occipital central regions at 20 – 30 Hz (slow beta), maximally at 26 Hz; Coherence Factor 10, with decreased connectivity for the E-group compared to the C-group in narrowly focused, short distance connectivities, likely indicating again over connectivity now between occipital and right, left and central parietal regions at 12 Hz (alpha) [14,40]; Coherence Factor 13, also with decreased coherence for the E-group compared to the C-group, narrowly focused, multiple small region connectivities between the left frontal and right prefrontal temporal, parietal and occipital regions and the left posterior parietal regions, at 10 Hz (alpha), and given the mixture of long and short distance connectivities in one factor, more difficult to interpret; as well as Coherence Factor 11, also with E-group decrease in connectivity compared to the C-group, of a very focused, very short distance and restricted to within one quadrant connectivity between the right prefrontal area and largely immediately adjacent electrodes, at 18–28 Hz (beta). Figure 6 displays these factors. On the basis of these factors only 2 subjects, 1 C- and 1 E-group child, were misclassified.

**Table 5:** Discriminant function analysis of EEG coherence factors

Jackknifed Classification* Matrix ***Coherence Factors 1,3,4,8,10,11,13,20***	**Correct Classification**	**Control** **(n = 12)**	**Experimental** **(n = 9)**
Control (C) Group	91.7%	11	1
Experimental (E) Group	88.9%	1	8
Total	90.5%	12	9

Wilks’ Lambda = 0.1561; *df* = 8,12; F = 8.11; *p =* 0.001. Jackknifed classification* efficiency is calculated by sequentially eliminating one case at a time, computing the discriminant function based on remaining subjects, and using the resultant discriminant to classify the eliminated case. Percent correct classification is based on the overall success of the total sample’s cumulative classification in this manner.

Overall, the factors that successfully discriminated E- from C-group subjects indicated a pattern of increased long distance, bi-hemispheric, across-midline connectivities from frontal to broad occipital as well as temporal and parietal regions (three factors) and of multiple decreased short and mid distance connectivities between more limited adjacent and more focused regions (five factors), as was hypothesized on the basis of the newborn IUGR studies that showed an overall similar coherence pattern. Thus, the NIDCAP intervention for this IUGR school-age population primarily appears to have increased long distance coupling between the areas of the brain which are associated with visual spatial, visual motor, visual planning and overall EF [16,78], and decreased possibly pathological over-connectivities between more immediately adjacent brain regions, an over-connectivity phenomenon that may well be due to the brain pathological consequences of the IUGR condition [14,40]. Findings were restricted mainly to the alpha and beta frequency bands, known to be involved in attention and mental processing.

### Brain structural outcome at school-age

**Figure 7:**
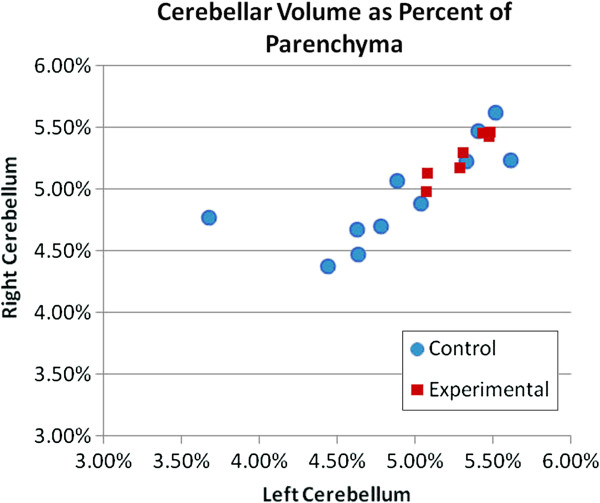
**Distribution of right and left cerebellar tissue volumes by group.** The figure represents a scatter plot of the control (n = 11) and the experimental (n = 7) groups’ distributions of right and left cerebellar tissue volumes expressed as percentage of total parenchyma.

Eighteen (11C; 7 E) of the 23 subjects had complete MRI studies. For three (2C; 1E) of the five subjects who did not have MRI studies, the parents were too hesitant to permit participation in the MRI. One child (E) had a medical implant that prevented her from participating in the MRI, and one child’s (C) data were not usable due to too much movement artifact. Of the two primary hypothesized brain structures and tissue types, one, namely bilateral frontal white matter (FWM), showed no significant volumetric group differences. In contrast, the second, namely the cerebellum, both right and left cerebellar hemispheres were significantly larger in the E-group children than the C-group children as was true for the total cerebellar volume with and without inclusion of the cerebellar mid-structure, the vermis (Table 6). Figure 7 shows the distribution of right and left cerebellar volumes for the control and the experimental group children. It indicates the tighter distribution around the mean for the experimental group in comparison to the control group, a phenomenon that was noted also for the neuropsychological measures. The E-group’s proportion of cerebellar volume to total parenchyma was comparable to that of a large normative comparison group while the C-group’s was significantly smaller [79]. None of the other structures measured for descriptive purposes showed any significant volumetric group differences.

**Table 6:** MRI tissue volumes

**Variable**	**Control**	**Experimental**	***p***
	(n = 11)	(n = 7)	
Right Cerebellum	4.91 (0.56)	5.31 (0.18)	0.05
Left Cerebellum	4.97 (0.36)	5.24 (0.18)	0.04
Total Cerebellum	9.88 (0.88)	10.55 (0.34)	0.04
Cerebellum + Vermis	10.65 (0.93)	11.38 (0.38)	0.04

Results are means (SD). Statistical analysis used is Brown-Forsythe Univariate Analysis of Variance F*, two-tailed. MRI tissue volumes were measured in cubic millimeters, per structure. To control for ventricular size, all measures were expressed as a percentage of total parenchyma which is total brain tissue adjusted for the ventricles.

Discriminant function analysis utilizing all 19 volumetric measures assessed, identified 6 measures, which significantly differentiated the C- from the E-group (Table 7). Brain structure volumes selected in the successful discrimination included again, as might be expected, the cerebellum inclusive of the vermis, as well as five other structures, namely brainstem, right caudate, occipital white matter, left nucleus accumbens, and left insula. Jackknifed classification success utilizing the 6 measures, showed 83.3% correct subject classification [74,75] (Wilks’ Lambda = 0.269; df = 6,11; p =0.01). Misclassified were 3 C subjects, while all E subjects were classified correctly. Thus, despite the further reduced subject number, the MRI measures were successful in differentiating C- from E-group children.

**Table 7:** Discriminant function analysis of MRI measures

Jackknifed Classification* Matrix ***Total Cerebellum, Brain Stem, Right Caudate, Occipital White Matter, Left Nucleus Accumbens, Left Insula***	**Correct Classification**	**Control** **(n = 11)**	**Experimental** **(n = 7)**
Control (C) Group	72.7%	8	3
Experimental (E) Group	100%	0	7
Total	83.3%	8	10

Wilks’ Lambda = 0.269; *df* = 6,11; F = 4.983; *p =* 0.01. Jackknifed classification* efficiency is calculated by sequentially eliminating one case at a time, computing the discriminant function based on remaining subjects, and using the resultant discriminant to classify the eliminated case. Percent correct classification is based on the overall success of the total sample’s cumulative classification in this manner.

### Classification success utilizing all three brain development domains

The 3 EF measures (Incidental Accuracy/Copy and Immediate Recall conditions, and Structural Accuracy/Copy condition), 8 EEG coherence factors (Factors 3, 20, 8, 4, 1, 10, 13 and 11), and 6 brain structure tissue volumes (cerebellum inclusive of vermis, brainstem, right caudate, occipital white matter, left nucleus accumbens and left insula), were examined as to their C- and E-group classification success in order to determine the relative power of each of the three brain development modalities. Discriminant function analysis utilizing these variables identified 6 measures, namely two coherence factors and four specific brain structure tissue volumes, which significantly differentiated the C- from the E-group (Table 8). Jackknifed classification success utilizing the 6 measures, showed 94.4% correct subject classification [74,75] (Wilks’ Lambda = 0.1699; df = 6,11; p = 0.001). Misclassified was only 1 C- subject. The variables included EEG Coherence Factors 3 again and Coherence Factor 20, as well as the brainstem, the right caudate, the left nucleus accumbens, and again the cerebellum inclusive of vermis. None of the EF measures was selected by the discriminant function analysis. The results indicated that the use of two brain development modalities, namely EEG and MRI, was more successful in discriminating the C- from the E- IUGR-preterm-born school-age children than measurement of one of the modalities alone. Classification was successful and highly significant despite the small sample size.

**Table 8:** Discriminant function analysis of Rey-Osterrieth, EEG and MRI measures

Jackknifed Classification* Matrix ***Total Cerebellum, Coherence Factor 3, Left Nucleus Accumbens, Right Caudate, Coherence Factor 20, Brain Stem***	**Correct Classification**	**Control** **(n = 11)**	**Experimental** **(n = 7)**
Control (C) Group	90.9%	10	1
Experimental (E) Group	100.0%	0	7
Total	94.4%	10	8

Wilks’ Lambda = 0.1699; *df* = 6,11; F = 8.96; *p =* 0.001. Jackknifed classification* efficiency is calculated by sequentially eliminating one case at a time, computing the discriminant function based on remaining subjects, and using the resultant discriminant to classify the eliminated case. Percent correct classification is based on the overall success of the total sample’s cumulative classification in this manner.

### Relationship between executive function and spectral coherence measures

Canonical correlation employed to explore the relationship of the 3 EF (ROCFT) measures (Incidental Accuracy/Copy and Immediate Recall conditions, as well as Structural Accuracy/Copy condition) and the 8 spectral coherence factors showed a significant relationship as identified with Bartlett’s test (χ^2^ = 38.01, df = 24, p < 0.035). The EF variable Incidental Accuracy from the Immediate Recall condition as well as Coherence Factor 3 and Coherence Factor 8 showed the highest correlations on the canonical variable. In summary, better visual spatial organization and memory as indexed by the Incidental Accuracy/Immediate Recall condition, was associated with stronger long distance connectivity between frontal, and broad occipital regions, and more diminished short distance connectivity between right and left occipital to parietal regions.

### Relationship of executive function and volumetric brain structure measures at school-age

Canonical correlation between the 3 EF measures (ROCF, Incidental Accuracy/Copy and Immediate Recall conditions, and Structural Accuracy/Copy condition) and the 6 Regional Volumetric Brain Measures (cerebellum inclusive of vermis, brainstem, right caudate, occipital white matter, left nucleus accumbens and left insula) were marginally significant (χ^2^ = 30.40, df = 21, p = 0.08), indicating a marginal association between EF and brain volumetric measures at school-age. Variables which loaded significantly on the canonical variate included ROCF Incidental Accuracy/Immediate Recall condition, (p = 0.001) and the MRI variable cerebellum inclusive of vermis (p = 0.02) suggesting a relationship between better EF function as measured by the ROCF and cerebellar volume.

### Relationships between neurobehavior at 2 weeks corrected age and executive function at school-age

Canonical correlation employed to explore the relationship between the six main APIB variables measured at 2 weeks corrected age [14], namely, Organization of the Autonomic, Motor, State, Attention, and Self-Regulation Systems as well as Degree of Facilitation required for systems reorganization, and the 9 ROCF EF variables, showed a significant relationship as identified with Bartlett’s test (χ^2^ = 102.49, df = 72, p < 0.01). The APIB measure, Attention System Organization in the newborn period and the Organization score in the Delayed Recall condition at school-age showed the highest correlations on the canonical variable. In summary, there appeared to be a significant relationship between the APIB attention measure in the newborn period and the ROCF EF measure at school-age denoting memory and visual spatial organization at school-age.

## Discussion

The results support the hypothesis that NIDCAP in the NICU enhances aspects of executive function underlying visual-motor and memory functions.

As newborns at 2wCA [14] the children who were in the NIDCAP-group showed significantly better neurobehavioral functioning as measured with the APIB [76] than did the control-group children as newborns. The correlation analysis on neurobehavioral functioning measures from the newborn to the school-age study point showed, in specific, that the newborns with better attention organization displayed better EF later as reflected in the Organization (i.e. Gestalt appreciation) score in the Delayed Recall (i.e. memory) condition of the Rey-Osterrieth Complex Figure Test [50].

The demonstrated superiority in executive functioning measures for E- versus C-group IUGR children in spite of academic and IQ equivalence is consistent with the results of other investigators, who suggest that despite considerable shared variability, the measures of executive functioning maintain unique variance that is not encapsulated in the construct of global intelligence [77]. The demands for the skill set considered under the general rubric of EF including planning, response inhibition, working memory, organizing and strategizing ongoing behavior increase significantly in middle and later school years when the child is increasingly required to monitor and self direct behavior. It may be hypothesized that the impact of EF differences may become increasingly apparent at a later age since it is reflected in measures of independent functioning and the development and maintenance of appropriate social relationships of increasing importance at later ages.

Electrophysiological function at school-age was also improved. Similar to the earlier findings for the IUGR sample in the newborn period (2wCA) [14] and its more recent replication study with a second IUGR sample [40], NIDCAP effects on EEG coherence resulted in a pattern of decreased short distance connectivity between multiple adjacent brain regions. However, it appears to have also resulted in increased long-distance, across-midline bilateral frontal to occipital connectivities in the E-group. This is similar to the pattern found in a comparable-in-gestational age AGA preterm sample in the newborn period [1] as well as an extremely early-born AGA sample at 8 years [16]. Without the experimental intervention in the preterm period the IUGR-preterm-born school-age children in the control group who did not receive experimental intervention in the preterm period show continued excessive cortical short-distance connectivities, possibly a carry-over from the multiple pathological influences generated by the IUGR condition. The IUGR children who received the NIDCAP intervention in the NICU appear to have preserved some of the plasticity evidenced in increased long-distance coherences in an AGA school-age sample, despite the IUGR condition. Presumably such connectivity is more conducive to better differentiated mental control function and visual motor integration [16,77,80]. In particular, Coherence factor 3, stronger in the IUGR E-group, appears to suggest increased connectivity between the dorso-lateral frontal cortex and bilateral occipital parietal regions. These regions are known to be active in visual spatial working memory [81-85] and thought to be largely independent of IQ [77]. Furthermore, better connectivity was correlated with better ROCF performance, an integrative visual spatial working memory task, in the IUGR E-group. Thus, the intervention appears to ameliorate excessive coupling noticeable still at school-age in the IUGR control group children, and to enhance cortical function in long distance connectivities supportive of differentiated attention regulation, visual spatial memory organization and executive function in the IUGR experimental group children. This finding of enhanced long distance cortical coupling and its implication for more effective executive functioning has been supported by others [16,78] who investigated whether varying demands on central executive processes are reflected by differences in coherence activity. Their results indicate that a decrease of anterior upper alpha short-range connectivity and a parallel increase of fronto-parietal long-range coherence may be considered as plausible candidates for the neural correlates of central executive function.

This is the first report of NIDCAP effectiveness at school-age for IUGR preterm-born children, and the first documentation of brain structural differences for IUGR preterm born children at school-age. The tighter distribution of right and left cerebellar volume measures around the group mean for the experimental as compared to the control group speaks not only to the greater symmetry of right and left cerebellum in the experimental group but also perhaps a better opportunity to develop than the control group experienced. The larger tissue volumes identified consistently for the right and left cerebellum are interpreted to reflect intervention-enhanced connectivity of the cerebellum with multiple other cortical regions [47,66,86-92] in particular frontal and prefrontal cortex [93] as in this study corroborated by the cortical coherence findings. The findings substantiate the cerebellum’s increasingly recognized, important role in complex behavior, including visual-spatial organization and complex integrative and executive function as exemplified in poor ROCFT performance [94,95] and cognitive and mental control functions [44,92,96].

Normative data [97] for a healthy sample (n = 433) of children from 4 to 18 years of age indicated mean cerebellar volumes of 132.82 cm^3^, which corresponded to 10.52% of brain parenchyma, comparable to the 10.55% found for the IUGR sample’s E-group while the IUGR C-group’s cerebellar volume was only 9.88%, a significant difference (p = 0.04).

Thus, the current study shows the cerebellum amenable to very early behavioral intervention during the time of its most rapid development [98]. The cerebellar findings are in keeping with the improved ROCFT findings and the coherence findings of enhanced broad frontal to broad occipital long distance connectivities observed in the experimental group. The results identified for the IUGR preterm born school-age sample are internally consistent and consistent with the most recent literature on the cerebellum and its functions. They are also consistent with two IUGR preterm studies in the newborn period. They speak to the effectiveness of the experimental intervention also for severely IUGR preterm infants who are already compromised in the womb with respect to the growth and function of multiple organs including brain. The results provide additional evidence for the effectiveness of the intervention delivered in the immediate newborn period. When the study children reached school-age, those who had been in the NIDCAP group demonstrated significantly better spatial visualization and mental control than those who received standard NICU care. The EEG-derived measures of cortical connectivity also successfully differentiated E-group from C-group children at school-age and corroborated the neuropsychological findings in terms of the neural pathways implicated. The findings, although limited by the small sample size, potentially have important implications for the amelioration of the specific learning disabilities with which so many early born IUGR children struggle. The study’s findings contribute to the validation of the hypothesis that underlies NIDCAP, namely that the fetal brain in the late second and throughout the third trimester is differentially sensitive to the repeated stressful events experienced in the NICU, and that this vulnerability may be compensated for by consistent individualized developmental care implementation. While the specific pathways involved in NIDCAP effectiveness are not fully understood, consistent implementation of individualized cue-based reintegration times specifically to support reliable re-synchronization of the autonomic, motor and state systems as well as attention organization appears to strengthen the infant’s self regulatory capacities. In the face of the many demands of intensive care, this individualized support appears to provide an opportunity for the immature brain to develop its connectivities in a more adaptive manner than is the case in a more traditional faster paced approach to intensive care. The reliable behavioral subsystem reintegration may provide protection of programmed cell death and of the development of better inter-cortical connectivities which may guard against compromised brain development. The neurobiological processes underlying NIDCAP effectiveness are speculative. It is postulated that NIDCAP processes may involve stabilization of the NMDA (*N*-methyl-d-aspartate) axis, protection against neurocytotoxic damage, lowered sensory and pain thresholds, and increased stability with modulated thresholds of reactivity and sensitivity [99-102]. Other potential mechanisms, inferred from results of differential mothering and sensory experience experiments in animal models, suggest that continuity and reliability of maternal care promotes hippocampal synaptogenesis as well as spatial learning and memory through systems known to mediate experience-dependent neural development [103,104], which appear to also include the cerebellum [66]. All of these effects may enhance NIDCAP group preterm infants’ capacity to take advantage of environmental offers in and beyond discharge from the stressful NICU environment.

In the NIDCAP model of caregiving, the infant’s behavior guides the caregiver in improving the infant’s comfort and in decreasing the infant’s stress. The NIDCAP approach focuses on individualization of care in the NICU as based on each infant’s behavioral cues, to support each infant’s strengths and to reduce the level of stress and pain experienced by the infant. Conscious, deliberate, and thoughtful caregiving and the consistent familiar presence of, and intimate contact with, the parents and other family members appears to support the infant to be more calm and self-regulated. This in turn appears to facilitate brain development [105-107]. This study provides the first indication that NIDCAP may demonstrate specific beneficial brain developmental effects for IUGR experimental group children not only in infancy but likely also into school-age; this is very encouraging.

Interpretation of the findings however requires much caution. The study’s most serious limitation is the small sample size. Further substantiation by larger longitudinal follow-up studies into school-age is necessary to corroborate the result of this preliminary, while promising, small study. Advances in newborn intensive care since the time of the NICU intervention phase of the study have implications for the interpretation of the results also. Although the exact underlying mechanisms of NIDCAP effectiveness remain to be discovered, evidence from this study points to the intervention’s possible long-term effectiveness. Future research is required to validate the encouraging results reported and to determine the intervention’s effectiveness beyond school-age into adolescence and early adulthood.

## Conclusion

Despite significant fetal compromise due to severe IUGR, preterm brain plasticity appears to prevail and specific brain based improvement that includes cerebellar improvement and consistent associated neuro-cognitive and spectral coherence findings appears to occur for the group that received the NIDCAP intervention in the NICU. Thus, NIDCAP, an individualized, behavior observation-based newborn intensive care and environment adaptation, appears to improve significantly the neurodevelopment of IUGR children with beneficial effects lasting into school-age in terms of behavior, functional brain connectivity and brain structure.

## Abbreviations

3D: Three dimensional; AGA: Appropriate in growth for gestational age; ANOVA: Analysis of variance; APIB: Assessment of preterm infants’ behavior; BESA: Brain electrical source analysis; BMDP: Biomedical data package; BR: Base rectangle; C: Control group; CA: Corrected age; CCAS: Cerebellar cognitive affective syndrome; CHIDDRC: Children’s hospital boston intellectual and developmental disabilities research center; df: Degree of freedom; DSC: Stepwise discriminate analysis; DSS-ROCF: Developmental scoring system; E: Experimental group receiving NIDCAP intervention; ECL: Eyes closed alert state; EDC: Expected date of confinement; EEG: Electroencephalography; EEGT: Electroencephalography technician; EF: Executive function; F: Frontal; F*: Browne-forsythe test of variance; FET: Fisher’s exact probability test; FWM: Frontal white matter; GA: Gestational age; GM: Grey matter; Hz: Hertz; IQ: Intelligence quotient; IUGR: Intrauterine growth restriction; KABC-II: Kaufman assessment battery for children, second edition; KBIT-2: Kaufman brief intelligence test, second edition; MPI: Mental processing index; MRI: Magnetic resonance imaging; MS: Main structures; N: Sample size; NICU: Newborn intensive care unit; NIDCAP: Newborn individualized developmental care and assessment program; NFI: NIDCAP federation international; NIH: National institutes of health; NMDA: *N*-methyl-d-aspartate; OWM: Occipital white matter; P: Probability values; PCA: Principal components analysis; R: Correlation coefficient; RCT: Randomized controlled trial; REEGT: Registered electroencephalography technician; ROCF: Rey-Osterrieth Complex Figure; ROCFT: Rey-Osterrieth Complex Figure Test; SD: Standard deviation; W: Weeks; WJ-III: Woodcock-Johnson III Tests of Achievement; WM: White matter; X¯ : Mean; χ2: Pearson chi-square; y: Years.

## Competing interest

The authors declare that they have no competing interests.

## Authors’ contributions

**GM**, as the manuscript’s first author, had a major role in the study’s design, developed the neuropsychological assessment battery, collaborated in the design of the data analysis, managed the coordination of all data acquisition and entry, performed the data analysis in collaboration with the study’s statistician (DZ) and the senior author (HA), and collaborated in interpretation of the results and the drafting and finalization of the manuscript. **FHD** collaborated in the study’s design, took the lead responsibility for the neurophysiological component of the study, developed, supervised and executed the electrophysiological data acquisition and performed their analyses, provided clinical referrals and follow-up as indicated, and collaborated in the interpretation of the results and the drafting and finalizing the manuscript. **SK** participated in the study’s design, managed all human subjects protocol aspects and renewals, obtained consent from participants and their parents, collaborated in the provision of an up-to-date literature review, designed and prepared all tables and figures and critically reviewed the manuscript. **NIW** and **SKW** participated in the study’s overall design, designed, developed, supervised and executed the magnetic resonance imaging data acquisition and analyses, and collaborated in interpretation of the results and the drafting and finalization of the manuscript. **SCB** participated in the study’s design, collaborated in the provision of an up-to-date literature review, collaborated in the coordination of data acquisition and entry, data abstraction from interview information, coding of test data and performance of data analysis, drafted result tables, and collaborated in the interpretation of the results, as well as the drafting and finalization of the manuscript. **MA** collaborated in all three domains’ (neuropsychological, electroencephalographic and magnetic resonance imaging) data acquisition, developed and problem-solved appropriate data acquisition strategies in order to assure timely and effective child participation, collaborated in the data base management, data reduction, coding and entry and the preliminary analysis of the pilot data collected for the study, and collaborated in the interpretation of the results as well as the drafting and finalization of the manuscript. **JHB** participated in the study’s design, collaborated in the development of the neuropsychological assessment battery, took responsibility for the Rey-Osterrieth Complex Figure Test scoring which she had originally developed, and for the interpretation of the results of this test, collaborated in the design of its data analysis, the interpretation of the results and the drafting and finalization of the manuscript. **RR** provided clinical review and report production of all magentic resonance imaging studies, collaborated in the provision of clinical referrals and follow-up as indicated, and contributed to the interpretation of the results and the drafting and finalization of the manuscript. **DZ** provided collaborative input to the study’s design; he provided the power calculations for the assurance of a sufficient sample size and the appropriate interpretation of results given the final sample size; he furthermore provided the design of the statsistical analysis and supervision of the data analysis, and he made contributions to the interpretation of the results and the drafting and finalization of the manuscript. **HA**, as the senior author, conceived of the study, participated in its design, the coordination and supervision of all data acquisition, assured the overall integrity of all aspects of the study including the continued blinding of all outcome assessors, supervised all aspects of the data analysis and interpretation of the results as well as the drafting and finalization of the manuscript. All authors read and approve the final manuscript.

## Authors’ information

The authors have collaborated for more than 15 years on a number of research studies on the effectiveness of the NIDCAP model of intervention in the newborn intensive care nursery on behalf of improved outcomes for preterm-born infants. This is the first collaborative effort in evaluating the school-age effects of the NIDCAP intervention for a very high risk preterm population, namely those with severe intrauterine growth restriction.

**GM** is Research Associate in Psychiatry at the Harvard Medical School (HMS) and Children’s Hospital Boston (CHB), as well as Associate Director of Neurobehavioral Infant and Child Studies in the Department of Psychiatry at CHB. She is a licensed neuropsychologist with special expertise in school-age neuropsychological assessment, as well as with graduate training and teaching experience and expertise in statistical design and data analysis.

**FHD** is a senior pediatric and adult neurophysiologist and neurologist at CHB, Associate Professor of Neurology at HMS and CHB, an international leader in quantified EEG and spectral coherence; he is well-versed in complex data analysis methods for quantitative EEG and its relationship to other data bases. He has published extensively on the applicability of spectral coherence in infant and child studies and has collaborated extensively with the first and the senior authors for more than 20 years. He is the Director of the Developmental Neurophysiology Laboratory at CHB.

**SK** is a many-year-experienced senior research coordinator with the Neurobehavioral Infant and Child Studies team. She managed all human subjects protocol aspects and renewals of the team for more than twenty years, including of this study; she is experienced in obtaining consent from participants and their parents, provision of up-to-date literature reviews, design and preparation of data tables and figures and in critical review of manuscripts.

**NIW** is a research fellow in image analysis with special expertise in preterm brain development. He has developed innovative algorithms for brain tissue segmentation and parcellation and is a gifted data manager of complex, large imaging data bases. His supervisor, **SKW** is Professor of Radiology at HMS and CHB, and Director of the Quantitative Imaging Laboratory, Department of Radiology, CHB and has collaborated on a number of research projects with the senior and the first authors as well as with FHD, the senior neurophysiology collaborator, for the last 10 years. He supervises a number of multi-disciplinary studies all involving magnetic imaging techniques and he is an internationally respected expert in innovative advances in the field of brain imaging.

**SCB** is a clinically licensed pediatric psychologist who has extensive training in developmental psychology and special expertise in the implementation of the NIDCAP intervention. She is a member of the Neurobehavioral Infant and Child Studies team in the Department of Psychiatry at CHB and holds the position of Instructor in Psychology (Psychiatry) at the HMS and CHB. She trained with the senior author in newborn research and assessment and has worked with the Neurobehavioral Infant and Child Studies team for the last ten years as an integral member of the research team.

**MA** is a licensed school psychologist with extensive experience in neuropsychological and school achievement assessment. Of all the co-authors she is the most recent addition to the Neurobehavioral Infant and Child Studies team in the Department of Psychiatry, CHB where she serves as independent assessor blinded to the newborn interventions in the intensive care nursery.

**JHB** is a clinically licensed senior neuropsychologist at CHB where she served for many years as the Director of the Neuropsychology Training Program and Director of the Neuropsychology Assessment Unit. She is Associate Professor of Psychology (Psychiatry) at HMS and CHB. She has special expertise in preterm children‘s long term development and she developed the developmental scoring system for the Rey Osterrieth Complex Figure Test (ROCFT), that was utilized in the reported study. She has published extensively on the ROCFT and its relevance for the assessment of preterm-born children as they grow up. She has collaborated with the senior author, the first author and the Neurology collaborator and co-author on a number of earlier studies on varying aspects of learning disabilities. She is an internationally highly respected teacher and collaborator in the area of neuropsychological development especially as concerns executive function and right hemisphere abilities.

**RR** is the Chief of Radiology at CHB and Full Professor of Radiology at HMS and CHB. He has collaborated and published with the research team for the last 20 years and has supervised a number of newborn studies directed by the senior author. His experience in the field of pediatric radiology and particularly brain imaging is of international renown.

**DZ** is a senior statistician at CHB and Assistant Professor in Biostatistics at HMS and CHB, with special expertise in complex multi-method clinical research studies. He has collaborated with the research team for over twenty years and has provided collaborative input to their studies’designs on many prior occasions.

**HA** is a clinically licensed psychologist with special expertise in the development of preterm-born infants. She is the Director of Neurobehavioral Infant and Child Studies at CHB and Associate Professor of Psychology (Psychiatry) at CHB and the HMS. She has published extensively on the effectiveness of the NIDCAP intervention methodology in improving early childhood outcome in preterm infants and most recently in IUGR preterm born infants. The current manuscript is the second manuscript she is co-authoring on school-age effects of NIDCAP. She is an internationally recognized expert on the assessment of preterm born infants and young children.

## Pre-publication history

The pre-publication history for this paper can be accessed here:

http://www.biomedcentral.com/1471-2431/13/25/prepub
